# Significance of maximum intensity projection technique of multimodal ultrasound imaging in differentiating follicular thyroid carcinoma from benign lesions

**DOI:** 10.3389/fonc.2024.1407611

**Published:** 2024-09-05

**Authors:** Wei Gao, Yutong Chen, Qiong Wu, Yi Li, Yuanyi Zheng, Yan Wang

**Affiliations:** Department of Ultrasound in Medicine, Shanghai Sixth People’s Hospital Affiliated to Shanghai Jiao Tong University School of Medicine, Shanghai, China

**Keywords:** thyroid follicular carcinoma, ultrasonography, contrast-enhanced ultrasonography, multimodal ultrasonography, maximum intensity projection

## Abstract

**Objective:**

Preoperative diagnosis for follicular thyroid cancer (FTC) remains challenging. The purpose of this study was to explore the maximum intensity projection (MIP) features, which can be utilized for reconstructing and characterizing the structure of microvascular in tissue, associated with FTC, and to explore the independent risk factors for FTC in combination with multimodal ultrasonography and blood indicators.

**Methods:**

This single-center, prospective, single-blind, observational study included patients with suspected follicular thyroid carcinoma based on preoperative ultrasonography findings. All patients underwent routine ultrasonography, contrast-enhanced ultrasonography (CEUS), and correlated blood indexes tests. Offline MIP reconstruction of the CEUS images was performed. The tumor was histologically diagnosed postoperatively. Multivariable logistics regression was utilized for analyzing MIP characteristics combined with multimodal ultrasonography and preoperative blood indicators to identify independent risk factors for FTC.

**Results:**

In this study, 61 thyroid nodules were finally included according to the atretic criteria. (1) Compared with traditional color profile ultrasonography and CEUS, MIP technology can provide more information regarding microvascular characteristics inside thyroid tumors. The short, rod-like, crossed, curved and firework-like features of MIP images revealed statistically significant differences between the benign and malignant groups. (2) Multivariable logistic regression analysis indicated that the firework-like MIP characteristics of microvascular, thyroglobulin (Tg) level and vessel intensity (VI) value were independent risk factors for malignancy.

**Conclusion:**

(1) MIP technology has potential applications in the differential diagnosis of follicular thyroid carcinoma from benign lesions. (2) Firework MIP microvascular characteristics, Tg values and VI values can serve as parameters for the differential diagnosis of follicular thyroid carcinoma from benign lesions. This study provides a novel approach idea for preoperative multimodal differentiation of follicular thyroid carcinoma from benign lesions.

## 1 Introduction

Follicular thyroid cancer (FTC), which is the second most common type of thyroid cancer (papillary thyroid carcinoma [PTC]), accounts for 10%–15% of all thyroid cancers (1) and easily spreads through blood, leading to distant metastasis (2). It has been reported that 15–27 percent of patients with FTC have already developed distant metastases upon initial diagnosis. Moreover, the risk of distant metastasis in FTC is 2–10 times that in PTC (3–5). The golden standard for diagnosing FTC vascular and/or tumor envelope invasion, which can be observed by continuous histopathological examination postoperatively (6). Fine–needle aspiration cytology (FNAC) cannot assess whether the tumor has invaded blood vessels or the capsule. However, despite obtaining a 2 cm long lesion tissue via coarse needle puncture biopsy, false negatives may occur due to the limitation of the sampling scope. The pathological results of these above two methods cannot distinguish the extent of tumor invasion into the capsule and blood vessels; therefor, FTC cannot be diagnosed (7). According to the guidelines (8) and common preoperative evaluation methods for thyroid nodules, including thyroid ultrasonography, serology and molecular diagnostic techniques, none of them can independently meet the needs of preoperative differentiation of follicular thyroid carcinoma from benign lesions (9, 10).

The occurrence and development of tumors are closely associated with neovascularization. Hence, evaluating of the microvascular in thyroid tumors may be an entry point for preoperative imaging differential diagnosis of FTC. Maximum intensity projection (MIP) can be utilized for reconstructing and charactering the structure of microvascular in tissues. This technique is based on high-resolution accumulation imaging technique of contrast-enhanced ultrasonography (CEUS). The MIP technique processes time-series images obtained during ultrasound contrast imaging by extracting the maximum intensity value for each pixel over the entire imaging duration, resulting in an integrated image. This method effectively reduces noise and highlights the dynamic changes and distribution characteristics of blood vessels which might enhance the ability to identify the characteristics of small blood vessels in follicular carcinoma (11). Li et al. utilized MIP technology for reconstructing breast CEUS at high frame frequency and revealed that that point-like, linear, or dendritic patterns were more common in benign tumors. Additionally, the tree pattern appears only in benign tumors. Whereas the crab-claw morphology is more common in malignant tumors, and the aforementioned phenomenon may provide new research ideas for follow-up studies on breast tumors (12). Superb microvascular imaging (SMI) is a new Doppler technology, that can display ultra-low-speed blood flow signals and maintain high resolution, frame rate and sensitivity (13–16). The vessel intensity (VI) value of the region of interest (ROI) can be obtained through this technology, reflecting the ratio of the blood flow signal in the ROI to the total number of color and gray pixels, thereby quantifying the blood flow information. Some studies revealed that SMI has some clinical value in differentiating benign and malignant thyroid nodules (17–19); however, the main type targeted in these studies was PTC. According to a study on microvascular density in FTC tissues, FTC has a higher microvascular density than that of PTC and FTA (follicular adenoma of thyroid gland), which may be related to its biological characteristic of easy transfer via the blood (17, 20). Evaluating all dimensions of blood flow in thyroid nodules is of great significance. Therefore, utilizing MIP to reconstruct microvascular in suspected follicular thyroid carcinoma angiography and SMI to measure VI in suspicious thyroid nodule may have potential applications value in differentiating follicular thyroid carcinoma from benign lesions.

Therefore, this study aimed to explore the MIP features associated with FTC, and identify independent risk factors for FTC in combination with multimodal ultrasonography and blood indicators.

## 2 Materials and methods

### 2.1 Patients

**Figure 1:**
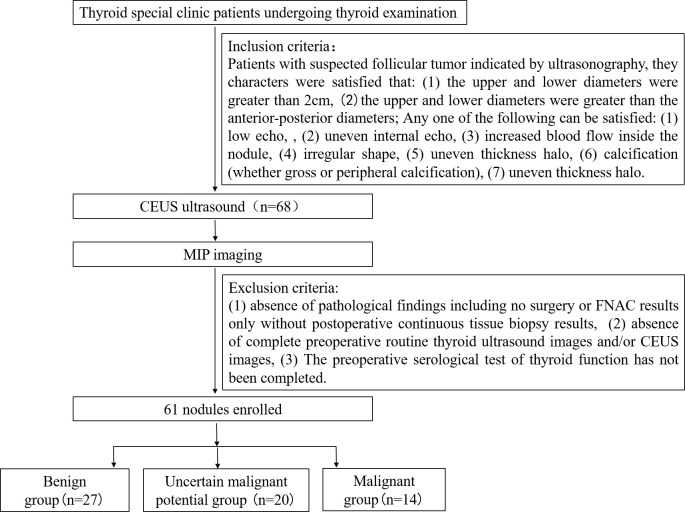
A flowchart presenting the recruitment of patients is shown.

This single-center, prospective, single-blind, observational study enrolled patients between January 2019 and December 2023 at the Thyroid clinic of Ultrasound Department. of Shanghai Sixth People’s Hospital, which accepted thyroid sonography patients referred from other medical centers or other doctors within the department. The inclusion criteria were as follows: patients with suspected follicular thyroid carcinoma indicated by ultrasonography meeting the following characteristics: (1) upper and lower diameters > 2 cm, and (2)upper and lower diameters greater than the anteroposterior diameters; and with any one of the following: (1) low echo, (2) uneven internal echo, (3) increased blood flow inside the nodule, (4) irregular shape, (5) uneven thickness halo, (6) calcification (gross or peripheral calcification), and (7) uneven thickness halo. The exclusion criteria were as follows: (1) absence of pathological findings including no surgery or FNAC results only without postoperative continuous tissue biopsy results; (2) absence of complete preoperative routine thyroid ultrasonography images and/or CEUS images; and (3) incomplete information for preoperative serological tests for thyroid function. A flowchart presenting the recruitment of patients is shown in **Figure 1**.

### 2.2 Research process

All enrolled patients underwent routine ultrasonography, high-frame-rate CEUS (Hi-CEUS), and CEUS. Both Hi-CEUS and conventional CEUS use sulfur hexafluoride microbubbles as contrast agents (SonoVue, Milan, Italy). The contrast medium concentration was ⅓ of the normal concentration, with a dose was 1.5ml, whereas conventional angiography was conducted at the usual concentration, with a dose of 1.2 mL. The primary endpoint of the study was the differences in morphological features of microvascular, as shown by MIP between the benign and malignant groups. The secondary end point combining the new MIP index with the old conventional indexes to determine the weight of the above index in differentiating follicular thyroid carcinoma from benign lesions. This study was approved by the Ethics Committee of Shanghai Sixth People’s Hospital (approval number: ChiCTR2100048361), and the research process strictly adhered with the Declaration of Helsinki. All patients signed an informed consent form during enrollment.

### 2.3 Conventional ultrasonography

The instruments used for routine ultrasonography included linear array probes equipped with Siemens Acuson S3000, Canon Aplio500, and Resonance 9 (Mindray, Shenzhen, China), at a frequency of 5-18MHz. experienced sonographers examined all patients. First, B-mode scan was conduct to measure tumor size (maximum diameter). Afterward, sonographers observed morphology (regular, irregular), physical properties (solid, mixed), internal echo (low echo, equal echo, high echo), boundary (clear, unclear), envelope exudation (yes, no), surrounding sound halo (yes, no), cystic change (yes, no), cystic change range (no, < 50%, ≥50%), calcification (yes, no), and aspect ratio. Secondly, color Doppler imaging was used to observe the blood flow (mainly internal, mainly peripheral, diffuse distribution). Finally, spectral Doppler imaging was used to measure the blood flow within the nodule [PSV (peak systolic velocity), EDV (end-diastolic velocity), RI (resistance index)].

### 2.4 CEUS

A linear array probe equipped with Resonance 9 (Mindray, Shenzhen, China) was utilized for scanning at a frequency of 5–18 MHz. First, Hi-CEUS images were collected. During the image collection process, patients were instructed to hold their breath for 10 s to prevent motion artifacts during subsequent MIP reconstruction due to respiratory activity. The Hi-CEUS mechanical index was 0.06, and the Hi-FR mode was used for angiography (the frame rate was generally > 40, varying with image depth to show the best lesion area and the focal point was positioned in the deepest part of the nodule). The contrast agent (SonoVue, Milan, Italy) was configured according to the manufacturer’s instructions. Afterward, 0.5 mL of SonoVue was extracted, and mixed with 1 mL of normal saline to create 1.5 m; then slowly injected through the anterior elbow vein. After injection, 5 mL normal saline was added to the tube or washing. The purpose of slowly injecting with low concentration is to allow bubbles to enter the area of interest gradually, to avoid instantaneous massive perfusion. This helps prevent failure in identifying single micro-bubbles during MIP reconstruction and affecting the presentation of tiny blood vessels. Simultaneously, 3 minutes of the video was recorded after injection.

After Hi-FR image acquisition, the micro-bubbles in the imaging area were exploded by clicking the FLASH button several times. Routine imaging video acquisition was performed when almost no contrast agent microbubbles were observed in the region of interest.

In routine CEUS, the instrument, patient preparation, and brand of contrast agent were the same as above; however, the concentration of the contrast agent was the original concentration, the dosage was 1.2 mL, and the injection method was intravenous mass injection, instead of slow administration.

The evaluation indexes of conventional CEUS analysis were recorded as follows: time of contrast agent entering the nodule (rapid entry, simultaneous entry, slow entry); way of entering the nodule (centripetal and non-centripetal including diffuse and centrifugal); intensity of enhancement at peak (high enhancement, equal enhancement, low enhancement), uniformity of intensity distribution of contrast macrovesicles at peak (uniform, uneven), early regression of contrast media in nodules (with or without early regression compared with adjacent thyroid parenchyma); regression rate (rapid regression, slow regression); presence of perfusion defect; range of perfusion defects (none, <50%, ≥50%); and presence of ring enhancement.

### 2.5 MIP imaging

All frames from the time when the first microbubble entered the lesion area to the time before the contrast agent reached its peak. The dynamic image data were processed offline using MATLAB R2023a software (MathWorks, USA), and one MIP image was generated every five frames, which were stacked to generate multiple MIP images. Images were saved in the corresponding fold using JPG format.

### 2.6 MIP image analysis and evaluation

Two senior sonographers evaluated the microvascular features of a series of MIP images from each case. In case of disagreements between two doctors, a third senior sonographer performed a reevaluation until reaching a final consensus. Upon evaluation, three physicians were withheld the pathological findings, laboratory results, and other imaging features. In the process pictures that clearly present the shape of microvascular, three corresponding characteristics were observed: 1) point-, parallel short rod-like pattern; 2) cross-, twisted-like pattern; and 3) firework-like pattern. Among them, punctate and parallel short rod shapes are defined as the punctate and parallel short rod shape tiny blood vessels; microvascular as crossed and twisted vessels when they were non-linear and crossed; and a microvascular emanating from a point and spreading out like a firework around it as “firework-like” blood vessel.

### 2.7 SMI and VI measurement

Canon Aplio500 color ultrasonic diagnostic instrument was used, with a planar linear array probe at a frequency of 5–14 MHz. The size of the sampling frame was adjusted to include the edge and depth of the maximum longitudinal section of the tumor and frequency of color-wall filtering. The range of the blood flow velocity scale was adjusted to 1.0-2.0 cm/s, and the color gain was adjusted to detect small blood flow without spilling, to clearly display small and low-velocity tumor vessels. For clear images, the cSMI mode was superimposed, the freeze key was pressed. Three frames of images with the most abundant blood flow were selected by playback, the focal area was mapped in the FREE STYLE mode, and the ROI of the VI analysis was determined. The software automatically generated the VI value, and three measurements were averaged to obtain the final VI value of each case.

### 2.8 Elasticity imaging

Hitachi Hi Vision peritus (Japan) was selected for elasticity imaging in the strain elastic imaging mode. The steps were as follows: sampling frame contained the nodule boundary, the sonographer held the probe and made steady frequency and small vibration perpendicular to the lesion, pressed the freeze button after stabilizing the image, and replayed a relatively clear and accurate frame to make a diagnosis. This process was repeated three times for identifying the relative accuracy of the elasticity score. Results of elastic imaging were evaluated according to a 5-points scales: 1 point, all lesions were green; 2 points, the focus is mainly green, mixed with a small amount of red; 3 points, The coverage area of green and red lesions was equivalent; 4 points, lesions were mainly red, and mixed with a small amount of green; and 5 points, all lesions were red.

### 2.9 Detection of serological markers

Preoperative serological marker tests included thyroglobulin (Tg), T3, T4, thyroid-stimulating hormone (TSH), TgAb, and thyroid peroxidase antibody (TPOAb) levels, and all blood samples were tested in our laboratory.

### 2.10 Statistical methods

The calculation of sample size was based on achieving the main study endpoint. Based on the preliminary experimental results, we estimated that the two indicators of crossed and curved blood vessels and firework characteristics may be valuable for the differential diagnosis of benign and malignant tumors. An alpha value of 0.5 and power value of 0.9 were used as the benchmark for sample size estimation. The minimum sample sizes required for the two indices was 53 and 24 cases, respectively. According to the principle of sample size estimation, the minimum sample size was 53 cases. Factoring in a 10% allowance lost/dropped cases, each group of lost/dropped patients accounted for approximately five cases; therefore, the final minimum sample size of each group was set at 58 cases.

Statistical analysis was conducted using IBM SPSS (version 26.0). Measurement data conforming to normal distribution are represented as mean ± standard deviation. An independent samples t-test was utilized for comparisons between two groups, one-way analysis of variance was used for comparisons across multiple groups. Median and quartile spacings were used to indicate that a normal distribution was not satisfied. The non-parametric Mann-Whitney U test was used for comparison between two groups, and the Kruskal-Wallis’s test was used for comparison between multiple groups. Count data are presented as frequencies and percentages. The chi-square test was used for comparisons between groups. When the number of cells with T<1 or 1<T<5 was greater than 1/5 of the total number of cells, fisher’s exact probability method was used to calculate it.

Subsequently, with pathological results as the dependent variable, the included nodules were categorized as benign, malignant, and with undetermined malignant potential. Possible statistically significant variables (*P* < 0.05) in the univariate analysis were selected as independent variables. Multivariable logistic regression analysis was conducted for benign, malignant, and undetermined malignant potential groups, and the independent risk factors related to the malignant group were screened. Statistical significance was set at *P* < 0.05.

## 3 Results

### 3.1 MIP characteristics and general clinical data analysis

Based on the inclusion criteria, 68 thyroid nodules were included. Among these, seven were excluded, as three cases were not treated surgically, and two cases only had FNA results without final histological and pathological reports, and two cases lacked complete information on preoperative serological test of thyroid function. Finally, 61 nodules were included, of which 27 were benign tumors, 14 were malignant tumors, and 20 were thyroid follicular tumors with uncertain malignant potential. The demographic characteristics of 61 patients are summarized in **Table 1**. The included nodules were categorized into three groups: benign (including follicular adenoma of the thyroid gland and, follicular nodular degeneration of the thyroid gland), malignant (FTC), and uncertain malignant potential (UMP) (eight with suspected local envelope infiltration). The clinical and MIP characteristics of the three groups are shown in **Table 1**. Among the study participants, the sex ratios of the three groups were 1:3.5, 1:2.5 and 1:1.5, respectively. No statistical difference was observed in sex distribution among the three groups (*P* > 0.05). Similarly, age distribution did not significantly differ among the three groups (44.56 ± 15.829 vs 49.50 ± 16.047 vs 46.50 ± 15.004 years; *P* > 0.05).

**Table 1:** Clinical data and MIP characteristics.

Variable	Benign	Malignant	FT-UMP	χ^2^/F	*P* value
Age (y)	44.56 ± 15.829	49.50 ± 16.047	46.50 ± 15.004	0.464	0.631
Sex				1.753	0.416
Male	6 (22.2)	4 (28.6)	8 (40.0)		
Female	21 (77.8)	10 (71.4)	12 (60.0)		
Point-like pattern				1.660	0.436
Absent	12 (44.4)	9 (64.3)	9 (45.0)		
Present	15 (55.6)	5 (35.7)	11 (55.0)		
**Parallel short rod-like pattern**				10.174	**0.004**
Absent	0 (0)	5 (35.7)	3 (15.0)		
Present	27 (100)	9 (64.3)	17 (85.0)		
Slightly twisted -like pattern				0.979	0.613
Absent	15 (55.6)	10 (71.4)	12 (60.0)		
Present	12 (44.4)	4 (28.6)	8 (40.0)		
**Cross-, curved-like pattern**				6.670	**0.036**
Absent	19 (70.4)	4 (28.6)	12 (60.0)		
Present	8 (29.6)	10 (71.4)	8 (40.0)		
**Firework-like pattern**				15.287	**<0.001**
Absent	24 (88.9)	4 (28.6)	13 (65.0)		
Present	3 (11.1)	10 (71.4)	7 (35.0)		

The bold values: P value<0.05.

**Figure 2:**
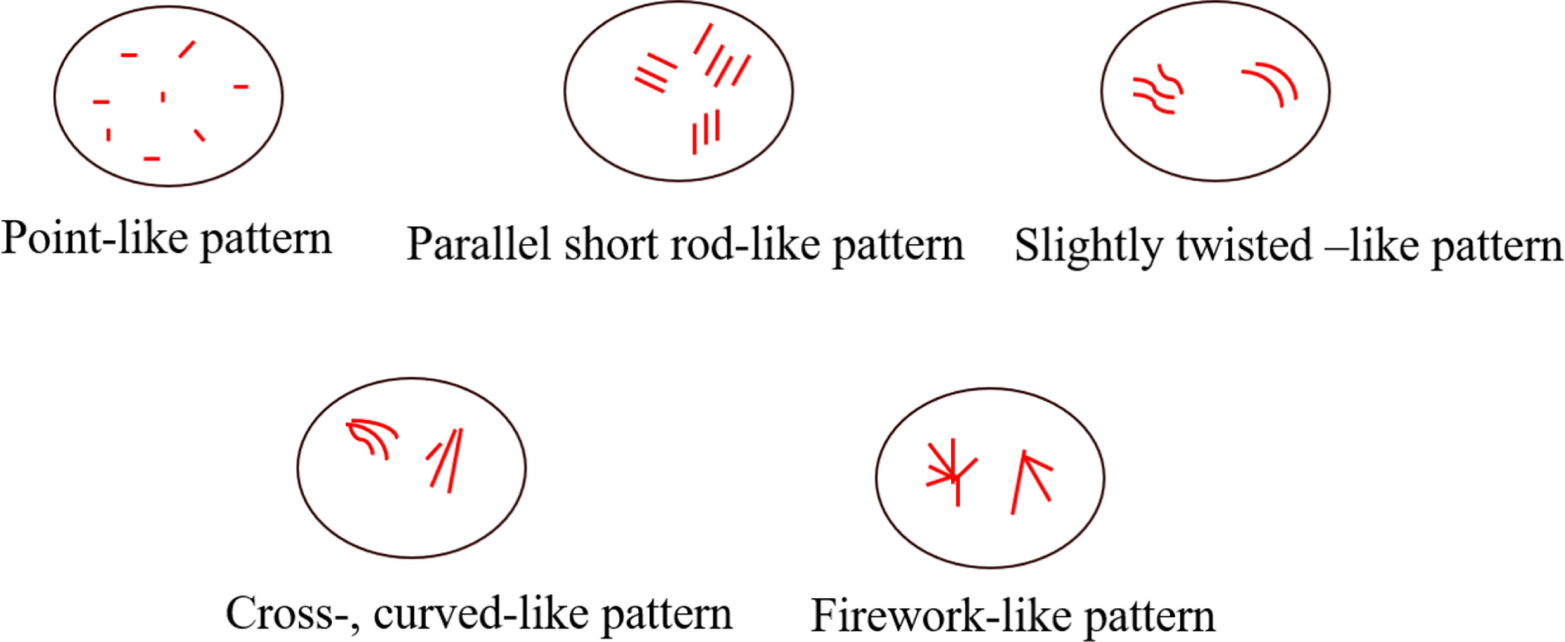
A diagram of the MIP features.

During gradual accumulation, the shape of the microvessels gradually appeared, in this process, and the typical characteristics of the microvessels were identified as follows: (1) point-like pattern; (2) parallel short rod-like pattern; (3) slightly twisted-like pattern; (4) cross, curved-like pattern; and (5) firework-like pattern. Correlation diagram is shown in **Figure 2**.

Based on these five characteristics, we statistically analyzed and registered the characteristics of the microvessels for each case, as shown in **Table 1**. Preliminary univariate analysis showed no statistical significance in the distribution of microvascular dots and slightly twisted-like pattern vessels (*P* > 0.05). In the short rod shape, crossed and curved -like pattern, and firework-like pattern (*P* < 0.05). We further compared the correlation of these indicators between the benign and the malignant groups, and found that the characteristics of fireworks-like pattern had the highest correlation with the malignant group (**Table 2**).

**Table 2:** Correlation between MIP characteristics and benign and malignant groups.

MIP characteristics	Correlation index	*P* value
Parallel short rod-like pattern	-0.365	0.004
Cross-, curved-like pattern	0.318	0.013
**Firework-like pattern**	0.449	**<0.001**

The bold values: P value<0.05.

**Figure 3:**
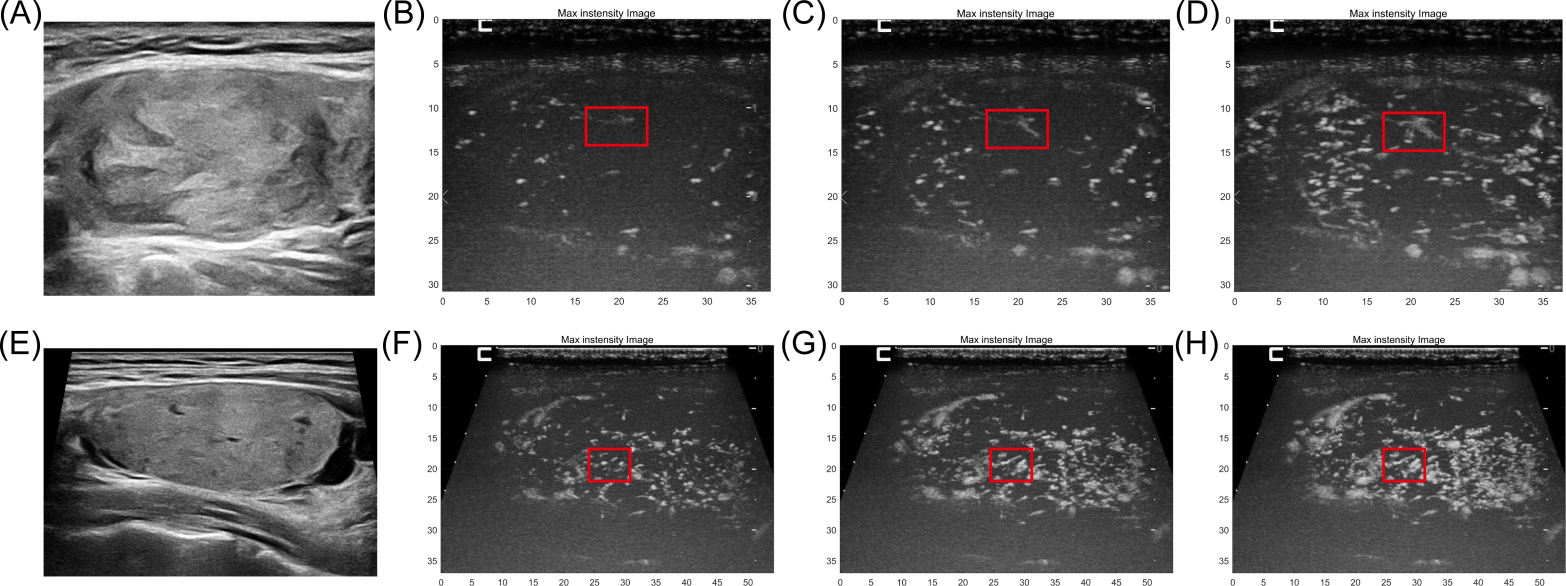
The conventional ultrasound and MIP image sequences of two cases of suspected follicular thyroid tumors with similar conventional ultrasonography characteristic. **(A)** B-mode ultrasound of a 44-year-old woman with FTC proven by pathology. **(B–D)** MIP sequences of FTC refer the microvascular with typical firework-like pattern characteristic (red box). **(E)** B-mode ultrasound of a 35-year-old man with FA proven by pathology. **(F–H)** MIP sequences of FA, microvasculature morphology shows typical parallel-, short rod-like pattern.

**Figure 3** shows conventional ultrasound and MIP image sequences of two cases of suspected follicular thyroid tumors with similar conventional ultrasonography characteristic. **Figures 3A–D** is a 44-year-old woman with FTC proven by pathology, and **Figures 3E–H** is a 35-year-old man with FA proven by pathology. **Figure 3A** shows a two-dimensional B-MODE image of FTC, **Figures 3B–D** illustrates the process of MIP sequence display gradually displaying the morphology of microvascular, and the red box refers the microvascular with typical firework-like pattern characteristic. **Figure 3E** illustrates a two-dimensional B-MODE image of a follicular adenoma (FA), and the red box in **Figures 3F–H** indicates the presentation of typical parallel-, short rod-like pattern characteristic.

**Figure 4:**
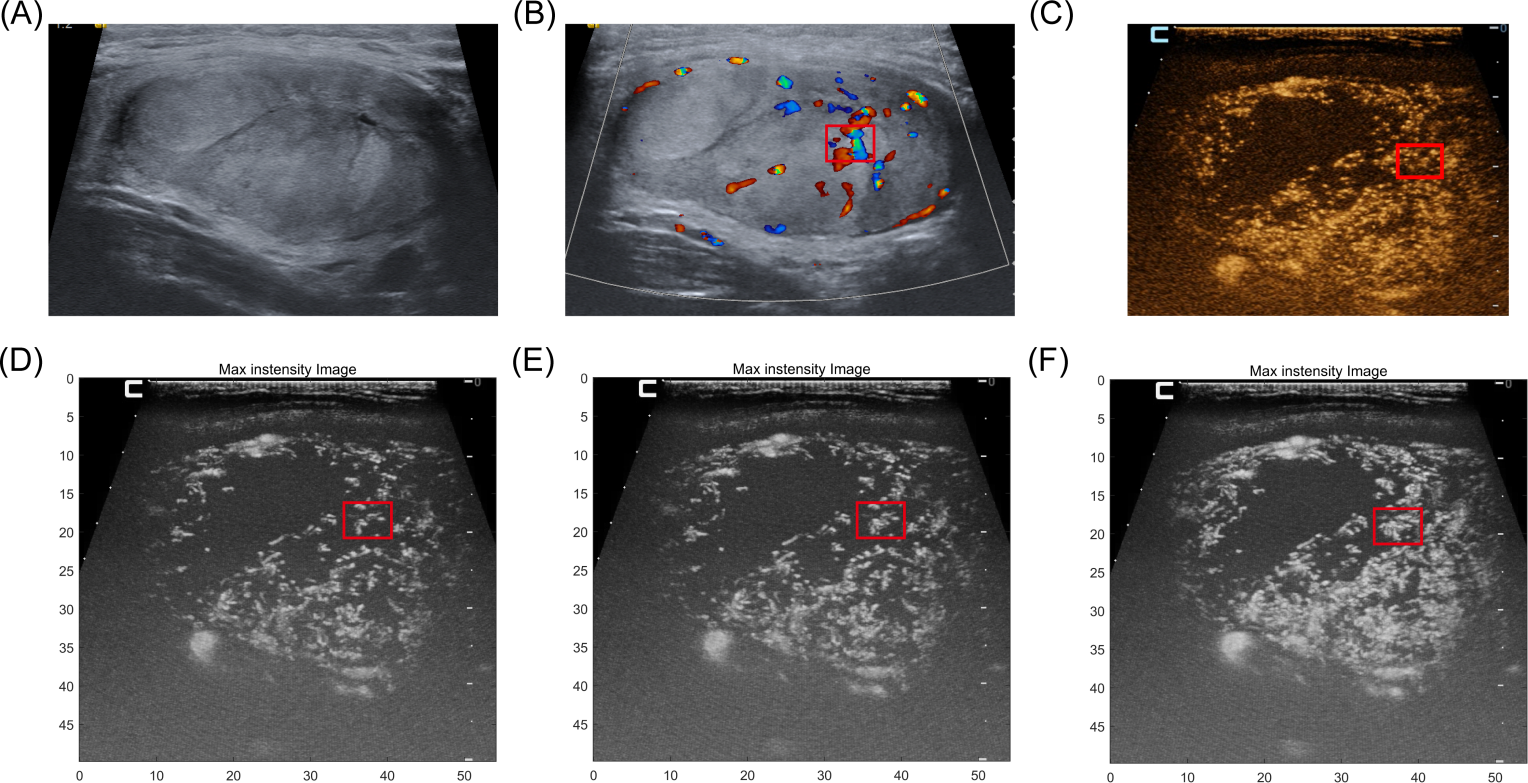
A 36-year-old man with FTC proven by pathology. **(A)** B-mode ultrasound of FTC. **(B)** color Doppler image of this case. only several thick blood vessels are displayed. **(C)** For the CEUS image, the microbubble just be record as a brief glimpse, the continuous vascular structure of the area is not displayed. **(D–F)** The microvascular shape with the typical firework-like feature, as shown in the red box but the information displayed can be continuously obtained by MIP sequences.

**Figure 4** depicts conventional ultrasound, color Doppler image, CEUS, and MIP sequence images of an FTC. The microvascular shape with the firework-like pattern feature, as shown in the red box in **Figures 4D–F**. However, the corresponding structure is not imprinted on the color Doppler image; instead, only several thick blood vessels are displayed. For the CEUS image, the microbubble just be record as a brief glimpse, but the information displayed by MIP can be continuously obtained.

### 3.2 Univariate analysis combined with multimodal ultrasonography

We conducted a preliminary univariate analysis of conventional ultrasonography features, CEUS features, VI values, and preoperative serological indicators of thyroid function, statistically significant differences were observed among all groups (*P* < 0.05) in annular blood flow, VI values, and Tg level (**Table 3**).

**Table 3:** Univariate analysis of conventional ultrasound characteristics, CEUS characteristics, VI value and preoperative serological indicators of thyroid function.

Variable	Benign	Malignant	FT-UMP	χ^2^/H (K)	*P* value
**Annular**				17.431	<**0.001**
blood flow					
Absent	10 (37.0)	14 (100)	15 (75.0)		
Present	17 (63.0)	0 (0)	5 (25.0)		
**VI value**	11.80 (6.30,18.50)	23.10 (16.475,25.20)	15.60 (12.175,28.95)	11.208	**0.004**
**Tg level**	53.90 (25.90,254.00)	554.45 (135.35,1585.75)	141.00 (45.51,453.00)	8.979	**0.011**

The bold values: P value<0.05.

### 3.3 Multivariable logistic regression analysis

Next, a multivariable logistic regression analysis was performed for the three classification groups (benign, malignant, and undetermined malignant potential). This included statistically significant variables obtained from each previous univariate analysis and indicators considered impactful by professionals. Considering the collinearity between independent variables, a stepwise forward regression was adopted, which include the following factors: annular blood flow, Tg level, and VI value of microblood flow, firework-like MIP feature, crossed and curved vessels MIP features, and short rod-like MIP feature (**Table 4**). Result indicated that upon comparing the malignant and benign groups (**Table 4**, group 1), the differences in the Tg level and, VI values and firework-like MIP characteristics were statistically significant. These parameters were considered independent risk factors for follicular thyroid carcinoma. For one-unit increase in Tg level, the probability of developing a malignant tumor increased by 1.003 times. Similarly, a one-unit increase in VI value corresponded to a 1.136-fold increase in malignancy probability. When comparing the malignant potential group with the benign group (**Table 4**, group 2), only the difference in VI was statistically significant.

**Table 4:** Multi-factor Logistics regression analysis.

Group	Variable	P value	OR Value	95%CI
1 (malignant group compared with the benign group)	Intercept	<0.001		
	VI value	0.019	1.136	1.021-1.263
	Tg level	0.018	1.003	1.000-1.005
	Annular blood flow (Absent)		379555029.077	379555029.077-379555029.077
	Firework-like pattern (Absent)	0.010	0.040	0.003-0.468
	Cross-, curved-like pattern (Absent)	0.256	0.257	0.025-2.678
	Parallel short rod-like pattern (absent)	0.998	130471035.281	
2 (malignant potential group compared with the benign group)	Intercept	0.074		
	VI value	0.017	1.136	1.023-1.261
	Tg level	0.140	1.001	1.000-1.003
	annular blood flow (Absent)	0.168	3.170	0.614-16.354
	Firework-like pattern (Absent)	0.196	0.270	0.037-1.962
	Cross-, curved-like pattern (Absent)	0.889	1.125	0.213-5.936
	Parallel short rod-like pattern (Absent)	0.998	79996688.144	

## 4 Discussion

This study prospectively included patients with suspected follicular thyroid carcinoma, as indicated by routine ultrasonography. MIP processing was conducted on the Hi-CUES video, and the morphological characteristics of microvascular within the tumors were analyzed and observed. It was revealed that the firework-like pattern of microvascular was an independent risk factor for FTC. Color Doppler ultrasonography and CEUS are real-time dynamic imaging techniques, that can only facilitate the identification the structure and perfusion of large blood vessels inside the tumor and have limited effect on the observation and evaluation of small and tiny blood vessels. According to previous studies, the value of these techniques in differentiating follicular thyroid carcinoma from benign lesions remains controversial. In this study, MIP technology was used to progressively superpose the morphological and structural characteristics of tiny blood vessels in tumors, providing a new diagnostic approach for differential diagnosis of follicular thyroid carcinoma from benign lesions.

In this study, we found the highest correlation with the malignant group when the microvascular morphology exhibited a firework-like pattern feature. In a study on the differential diagnosis of benign and malignant breast tumors using MIP technology, the “crab-claw” microvascular morphology was identified as a unique feature of malignant breast tumors (12), consistent with the results of the present study. During tumor occurrence and development, the construction and remodeling of new blood vessels remain crucial, with different characteristics in benign and malignant tumors. Most new blood vessels in malignant tumors exhibits invasive growth. The ischemic and hypoxic microenvironment due to rapid tumor proliferation results in the closure of most new blood vessels. Additionally, the micro-cancer embolism in the tumor leads to the embolization of new small blood vessels, making the small blood vessels in malignant tumors to be chaotic, invasive, and with sutural growth in all directions (21–23). This theoretical basis explains the firework-like pattern tiny blood vessels observed in our study, radiating from a central point in all directions. Unlike conventional ultrasonography and CEUS, which mainly display the extensive blood vessels and perfusion inside tumors, the anatomical and morphological characteristics of tiny blood vessels revealed in this study offer a new perspective for differentiating benign and malignant tumors.

MIP only reflects a single feature of microvascular characteristic; therefore, combining it with other indicators is crucial for improving the differential diagnosis of follicular thyroid carcinoma from benign lesions. Univariate analysis of conventional ultrasonography, CEUS and preoperative thyroid function serology revealed statistically significant differences in annular blood flow identified on conventional VI value measured by SMI and serum Tg level between groups. Moreover, multivariable logistic regression analysis revealed that the multiplication of VI level and serum Tg values was correlated with the probability of FTC occurrence. Benign thyroid tumors typically grow expansively, pushing blood vessels to the periphery, resulting in mainly peripheral or circular blood flow signals. Conversely, the blood flow signals of malignant tumors are mostly internal, out of shape, and exhibit a complex distribution (24, 25). Therefore, the difference in the index of circular blood flow in conventional ultrasonography in this study was statistically significant and consistent with the results of previous studies (26). A study on microvascular density in FTC tissues reveal that FTC has a higher density than that of FA (17, 20). Therefore, VI, which can reflect the microvascular density in tumor tissues, was included in this study as a research factor, and the results showed statistically significant component differences consistent with the results of previous studies (18). Tg is synthesized by thyroid follicular epithelial cells and stored in the follicular glia of the thyroid gland. When the follicular epithelium is damaged, high level of Tg are released into the blood. Serum Tg levels of patients is positively correlate with the risk of thyroid cancer, which is more significant in patients with FTC (27, 28). Increased serum Tg levels have been reported as an independent risk factor for FTC (29, 30). We also found significant differences in Tg levels between the groups.

This study has some limitations. First, owing to the low incidence of FTC, a short study period, and the collection of cases only from a single center, the number of included cases was small. Thus, multicenter studies with long-term follow-up can be conducted to include a larger and more diverse sample size to achieve greater accuracy. Nonetheless, despite the small sample size, the cases included in this study are extremely representative, due to obvious characteristics of suspicious thyroid follicular tumors on routine ultrasonographic images; however. they could not be differentiated as benign or malignant. Even when employing conventional thyroid nodule risk stratification systems (such as TI-RADS, C-TI-RADS, or K-TI-RADS risk stratification system), the risk of malignancy may be underestimated, favoring benign tumor diagnoses and leading to missed diagnosis (31). Moreover, patients with typical PTC were not included in this study. This decision was based on several characteristic indicators in routine ultrasonography, which can effectively screen for PTC and allow for definitive diagnosis via FNA preoperatively. Excluding such cases helped to minimize unnecessary research costs. Therefore, the model characteristics proposed in this study are suitable for the challenging differential diagnosis of follicular thyroid carcinoma from benign lesions. Second, the analysis and evaluation of MIP images were also subjective. Although we already recognized this issue and employed three doctors to analyze the images independently to balance the uncertainty brought about by the subjective evaluation, there is still a need for more objective and quantitative evaluation indicators. Furthermore, considering the heavy workload associated with manual reading of the images, evaluating MIP images becomes essential. Therefore, future research direction for our group involves developing an artificial intelligence prediction model for MIP images. Third, for the VI values and Tg levels, critical value or relevant results were not obtained for diagnostic efficacy evaluation. Therefore, future binary statistical studies can be conducted on benign group and malignant groups; the cutoff value can be obtained via statistical analysis of relevant dose data; and the sensitivity, specificity, area under the curve, and other indicators of diagnostic efficacy can be evaluated using the test set. To verify the efficacy of differential diagnosis between benign and malignant tumors, we classified the cases into three groups: benign, malignant, and undetermined malignant potential groups. Although we classified the undetermined malignant potential group as a separate group, we did not show the unique characteristics of this group. In previous studies, thyroid tumors with uncertain malignant potential (FT-UMP) were often classified as non-malignant along with the benign group due to their low likelihood of metastasis (32, 33). However, a follow-up study of 339 patients with FT-UMP in Japan revealed that although it is a low-grade malignant tumor, 1% of patients presented with recurrence and distant metastasis and received radioactive iodine therapy and orthopedic surgery (34); therefore, proper follow-up management of the patients is crucial. We suggested that FT-UMP should be considered as a separate research group. However, due to the limited duration of this study, long-term metastasis in these cases should be monitored in future studies, and the correlation between MIP characteristics and postoperative micro angiogenesis-related immunohistochemical indicators should be analyzed, to provide more biased and instructive suggestions for the postoperative management of these cases.

In summary, the MIP technique facilitates the reconstruction of microvascular inside thyroid nodule and can be used to identify differences in morphological characteristics of microvascular in benign and malignant nodules, providing a new diagnostic approach for preoperative multimodal differentiation of follicular thyroid carcinoma from benign lesions.

## Data Availability

The original contributions presented in the study are included in the article/supplementary material. Further inquiries can be directed to the corresponding authors.
